# Bringing the well being and patient safety research agenda together: why healthy HPs equal safe patients

**DOI:** 10.3389/fpsyg.2015.00211

**Published:** 2015-02-27

**Authors:** Efharis Panagopoulou, Anthony J. Montgomery, Evangelia Tsiga

**Affiliations:** ^1^Laboratory of Hygiene, Aristotle Medical School, Aristotle University of ThessalonikiThessaloniki, Greece; ^2^Department of Education and Social Policy, University of MacedoniaThessaloniki, Greece

**Keywords:** burnout, patient safety, employee health, health policy, organizational psychology

Health care is changing. Ageing populations, new therapeutic possibilities and rising expectations have made the provision of health care much more complex than in the past. The changing healthcare landscape means a greater burden for the healthcare professionals (HPs) who are expected to deliver the same quality of care with decreasing resources, while patient expectations of care remain stable or increase. Many countries in Europe are responding to this challenge by introducing new ways of delivering healthcare. However, the constant evolution of healthcare models is not resulting in better HPs, as indicated by the increasing phenomenon of burnout among health professionals (Leiter and Harvie, 1996; Rosenberg and Pace, 2006), or in safer care, as indicated by the increasing number of medical errors (Kondro, 2010). Today, there is enough evidence to suggest that expecting health professionals to deliver safe, efficient and patient-centered care, while they are getting more and more burnt-out, is not only ineffective but also costly and dangerous. In order for healthcare systems to be truly patient-centered, safe, and efficient, they need primarily to protect the health and well-being of their workers. Both healthcare professionals and patients are reinforced to view hospitals via a pathogenic lens. However, a saultogenic approach is needed. Interventions need to be bottom-up and system focused. Action research (AR) represents an appropriate methodology to link healthcare/patient input to improving hospital safety.

## HP and patient health entwined

One of the biggest risks for both patient safety and decreased quality of care today is health professionals themselves. Healthcare systems across Europe have systematically highlighted the importance of treating patients as whole people, not just as diseases, but health professionals (HPs) seem to be exempt from this holistic view. The European and Global trend is that between one third to half of health professionals are either feeling ill while present at work (i.e., presenteeism), or feel disengaged and demotivated (i.e., burnout) (Shanafelt et al., 2012). Substantial differences in burnout are observed by specialty, with the highest rates among HPs at the front line of care access (family medicine, internal, and emergency medicine). The consequences for HPs are significant and include; broken relationships, problematic alcohol use and suicidal ideation (Shanafelt et al., 2011; Oreskovich et al., 2012). The multi-center ORCAB (2014) project (http://orcab.web.auth.gr/) has recently shown that burnt-out HPs are at higher risk for medical mistakes. It has also highlighted that violence against HPs is increasing across Europe, especially women (European Foundation for the Improvement of Working and Living Conditions, 2010), and is driven by patient expectations and overburdened HPs. The aforementioned is exacerbated by increased patient expectations. Thus, a vicious cycle is created where inappropriate patient demands fuel feelings of exhaustion and depersonalization among HPs, which results in poor communication, which in turn ramps up already frustrated patient demands (and the cycle spirals downward).

## Healthcare reforms have overlooked the health of health professionals

The inability to reap the full benefits from current investments in health care results, in many instances, from the difficulties of creating and maintaining an effective, efficient and motivated workforce. At the same time, all attempts to develop a European platform of objective indicators for assessment of health care have not included indicators related to HP burnout and well being. For example, the Euro Health Consumer Index (EHCI) 2013 is the seventh study conducted on European healthcare systems. The Index takes a consumer and patient perspective, and includes the following indicators: Patient rights and information, accessibility, outcome, range and reach of services provided, prevention, and pharmaceuticals. Yet the EHCI, similarly to the previous studies have not included indicators on HP well being. The aforementioned represents a system that is self-perpetuating and quite likely to continue, whereby small “fixes” mask the more fundamental change.

## Policy making does not include enough evidence from frontline staff

Both in the US and Europe, evidence indicates that top-down approaches dominate in healthcare. Healthcare initiatives, changes or regulations are mainly developed by management boards, or policy making bodies with very little input from frontline staff or patients. This is also due to the fact that no systematic bottom-up feedback mechanism exists between healthcare delivery and policy making. As a result the effectiveness of initiatives adopted through the top-bottom approach is limited. Even more worrying is the fact that physicians responsible for delivering care are not interested in contributing to design, policy or evaluation. For example, the majority of US physicians do not feel responsible for reducing healthcare costs (Tilburt et al., 2013), and interventions are dominated by nurse-led initiatives, while physician examples are seriously lacking (Montgomery et al., 2015).

## The changes that are needed

**Figure 1:**
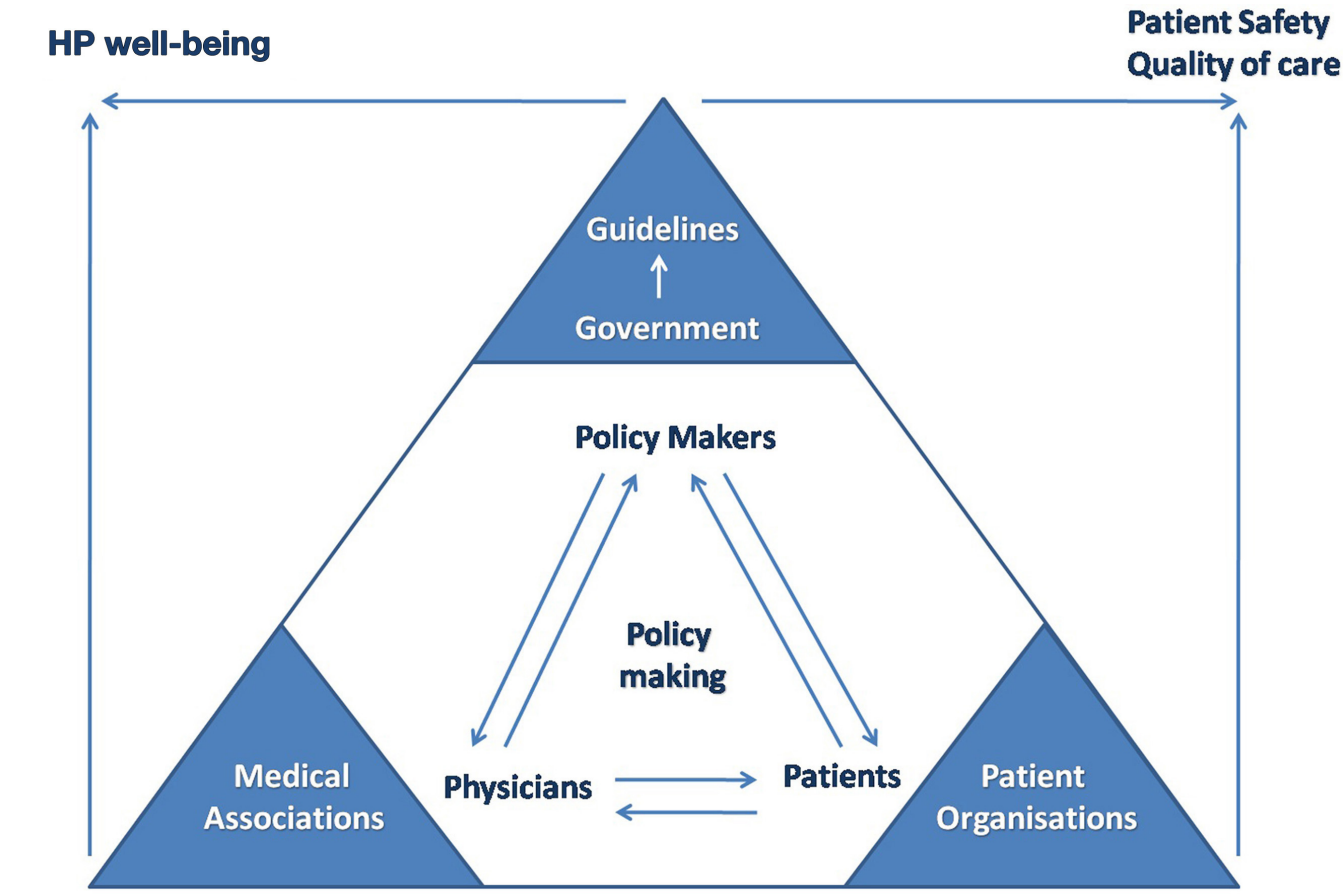
**Linking HP well-being and patient safety agenda**.

Medical associations have long been established with the goal of protecting, and supporting HPs in their professional role. Patient advocate organizations aim at supporting patients and promoting patient rights. Despite the fact that the ultimate goal of both types of organizations is to safeguard the quality of care, they still operate in parallel, or even against each other. We propose that Public, Patient, Professional Coalitions (3PCs) as an alternative governance mechanism, encouraging the participation of frontline staff, and patients, who have so far been largely sidelined from decision making in healthcare. For example, traditionally, patients contribute to service delivery via satisfaction surveys. We need to go beyond satisfaction surveys and involve the patients/public in rating indicators that are more directly linked to quality of care (e.g., presenteeism among HPs). There is considerable patient involvement in the planning and delivery of health services, but little evidence of the effect of this on quality and effectiveness of services (Crawford et al., 2002). Moreover, recent research indicates that there is currently insufficient evidence to be confident that the implementation of patient decision support interventions leads to system-wide savings (Walsh et al., 2014). This is more evidence of parallel (not integrated) tracks. The aforementioned is represented graphically in Figure 1.

## Conclusion

Interventions linking staff and patient health to better safety will fail if they don't challenge established ways of behaving. AR, unlike other research approaches aimed at generating knowledge, focuses on facilitating action and generating knowledge about that action.

### Conflict of interest statement

The authors declare that the research was conducted in the absence of any commercial or financial relationships that could be construed as a potential conflict of interest.
